# Web-Based Dietary Intake Estimation to Assess the Reproducibility and Relative Validity of the EatWellQ8 Food Frequency Questionnaire: Validation Study

**DOI:** 10.2196/13591

**Published:** 2021-03-02

**Authors:** Balqees Alawadhi, Rosalind Fallaize, Rodrigo Zenun Franco, Faustina Hwang, Julie Lovegrove

**Affiliations:** 1 Hugh Sinclair Unit of Human Nutrition and Institute for Cardiovascular and Metabolic Research, Department of Food and Nutritional Sciences University of Reading Reading United Kingdom; 2 School of Life and Medical Science University of Hertfordshire Hertfordshire United Kingdom; 3 Biomedical Engineering Section, School of Biological Sciences University of Reading Reading United Kingdom

**Keywords:** web-based, Kuwait, weighed food record, app, food frequency questionnaire, validation, dietary assessment

## Abstract

**Background:**

The web-based EatWellQ8 food frequency questionnaire (FFQ) was developed as a dietary assessment tool for healthy adults in Kuwait. Validation against reliable instruments and assessment of its reproducibility are required to ensure the accuracy of the EatWellQ8 FFQ in computing nutrient intake.

**Objective:**

This study aims to assess the reproducibility and relative validity of the EatWellQ8 146-item FFQ, which included images of food portion sizes based on *The Composition of Foods* by McCance and Widdowson and food composition tables from Kuwait and the Kingdom of Bahrain, against a paper-based FFQ (PFFQ) and a 4-day weighed food record (WFR).

**Methods:**

Reproducibility of the EatWellQ8 FFQ was assessed using a test-retest methodology. Participants were required to complete the FFQ at 2 time points, 4 weeks apart. To assess the relative validity of the EatWellQ8 FFQ, a subset of the participants were asked to complete a PFFQ or a 4-day WFR 1 week after the administration of the EatWellQ8 FFQ. The level of agreement between nutrient and food group intakes was estimated by repeated EatWellQ8 FFQ administration. The EatWellQ8 FFQ, PFFQ, and 4-day WFR were also evaluated using the Bland-Altman methodology and classified into quartiles of daily intake. Crude unadjusted correlation coefficients were also calculated for nutrients and food groups.

**Results:**

A total of 99 Kuwaiti participants (64/99, 65% female and 35/99, 35% male) completed the study—53 participated in the reproducibility study and the 4-day WFR validity study (mean age 37.1 years, SD 9.9) and 46 participated in the PFFQ validity study (mean age 36.2 years, SD 8.3). Crude unadjusted correlations for repeated EatWellQ8 FFQs ranged from 0.37 to 0.93 (mean *r*=0.67, SD 0.14; 95% CI 0.11-0.95) for nutrients and food groups (*P*=.01). Mean cross-classification into *exact agreement plus adjacent* was 88% for nutrient intakes and 86% for food groups, and Bland-Altman plots showed good agreement for energy-adjusted macronutrient intakes. The association between the EatWellQ8 FFQ and PFFQ varied, with crude unadjusted correlations ranging from 0.42 to 0.73 (mean *r*=0.46, SD 0.12; 95% CI −0.02 to 0.84; *P*=.046). Mean cross-classification into *exact agreement plus adjacent* was 84% for nutrient intake and 74% for food groups. Bland-Altman plots showed moderate agreement for both energy and energy-controlled nutrient intakes. Crude unadjusted correlations for the EatWellQ8 FFQ and the 4-day WFR ranged from 0.40 to 0.88 (mean *r*=0.58, SD 0.13; 95% CI 0.01-0.58; *P*=.01). Mean cross-classification into *exact agreement plus adjacent* was 85% for nutrient intake and 83% for food groups. Bland-Altman plots showed moderate agreement for energy-adjusted macronutrient intakes.

**Conclusions:**

The results indicate that the web-based EatWellQ8 FFQ is reproducible for assessing nutrient and food group intake and has moderate agreement compared with a PFFQ and a 4-day WFR for measuring energy and nutrient intakes.

## Introduction

### Background

According to the World Health Organization, noncommunicable diseases (NCDs) remain to be the main cause of global premature mortality [1]. Diets rich in energy and saturated fat and low in fruits and vegetables have been associated with the development of NCDs [2,3]. Inaccurate dietary assessment methods may be a serious obstacle in understanding the impact of dietary factors on disease [4]. Several dietary assessment methods are available, including the food frequency questionnaire (FFQ), diet history, weighed food record (WFR), and 24-hour dietary recall [5]. FFQs require respondents to state the frequency of intake of a predefined list of foods over a specified period and are one of the most commonly used tools to assess the relationship between diet, health, and disease [6].

With the widespread availability of the internet, there has been a growing interest in using the web to assess dietary intake and deliver health-related messages. Traditional dietary assessment methods have been customized for internet use in research as they allow for the direct storage of data and automatic generation of nutrition outputs [7,8]. In addition, web-based dietary assessment methods may be more cost-effective and can include photographs of food portion sizes, increasing the ease of use for respondents, and can be designed to be user-friendly and tailored toward a specific target group [9,10].

This study is part of the EatWellQ8 study, which aims to investigate whether web-based personalized nutrition (PN; based on dietary intake and anthropometrics) is as effective as face-to-face communication of PN in Kuwait. Kuwait currently has the highest adult obesity levels in the Gulf region [11]. The latest findings indicate that around 78% of adult men and 82% of women in Kuwait are either overweight or obese [12].

The novel EatWellQ8 FFQ was developed to assess the dietary intake in Kuwait and included 146 food items and photographs of food portion sizes. The validated Food4Me FFQ, the European Prospective Investigation of Cancer (EPIC) Norfolk FFQ (version CAMB/PQ/6/1205), and a paper-based FFQ (PFFQ) for Kuwait were used as guides in the development of the EatWellQ8 FFQ food items and categories of food [13-16]. Good agreement between the web-based Food4Me FFQ and the EPIC-Norfolk FFQ for the estimation of energy-adjusted nutrient intake was shown earlier [15,16].

### Objectives

The aim of this study is to develop and test the reproducibility of the EatWellQ8 FFQ for the assessment of food and nutrient intake in a Kuwaiti population for use in the EatWellQ8 study and to compare estimates of dietary intake using this tool with data obtained from a 4-day WFR and a validated paper, Kuwaiti FFQ (PFFQ) [14].

## Methods

### Study Sample

A sample size between 50 and 100 is recommended to accurately evaluate the Bland-Altman limits of agreement (LOA) between 2 methods [5]. Participants aged 18 to 65 years were recruited from Kuwait through email, poster advertisement, word of mouth, booths at colleges and health institutions, and social media (WhatsApp, Facebook, YouTube, and Instagram). Participants were then provided with an information sheet clarifying the study, a consent form, or an assent form (for participants aged 18-21 years) and asked to complete a web-based screening questionnaire. Participants were emailed a feedback response dependent on whether they met the inclusion criteria. A minimal set of exclusion criteria were applied (subjects aged below 18 years; pregnant or lactating; no or limited access to the internet; following a prescribed diet, including a weight-reducing diet in the previous 3 months; diabetes; celiac disease; Crohn disease; and previous chronic medical conditions requiring continuing therapeutic intervention apart from hypertension medication and statins). The study was approved by the Research Ethics Committee at the University of Reading (School of Chemistry, Food, and Pharmacy Research Ethics Committee, Ref. No. 13/17) and conformed with the Declaration of Helsinki. The study also received ethical approval from the Research Ethics Committee at the Dasman Diabetes Institute (DDI), Kuwait (RA-2015-018).

### Study Design

To assess the reproducibility of the EatWellQ8 FFQ, 100 participants were asked to complete the web-based FFQ twice, 4 weeks apart for intake over the past month, between the months of September and December. To assess the relative validity of the EatWellQ8 FFQ, participants were also asked to complete a 4-day WFR, a week after completing the web-based FFQ. An additional 50 participants were asked to take the EatWellQ8 FFQ at baseline and to complete a validated PFFQ for Kuwait a week after completing the web-based FFQ. The Kuwaiti PFFQ and the 4-day WFR were delivered to the participants in person or sent via email, depending on the participant’s preference. Participants were asked to complete the forms and hand them in person or to scan and email them to the researcher. Participants were asked to complete a usability survey after completing the first EatWellQ8 FFQ [17]. Reminders were sent biweekly to participants in the form of email and text messages to encourage completion of the tools. All participants were requested to maintain their usual diet during the study.

### The EatWellQ8 FFQ

**Figure 1:**
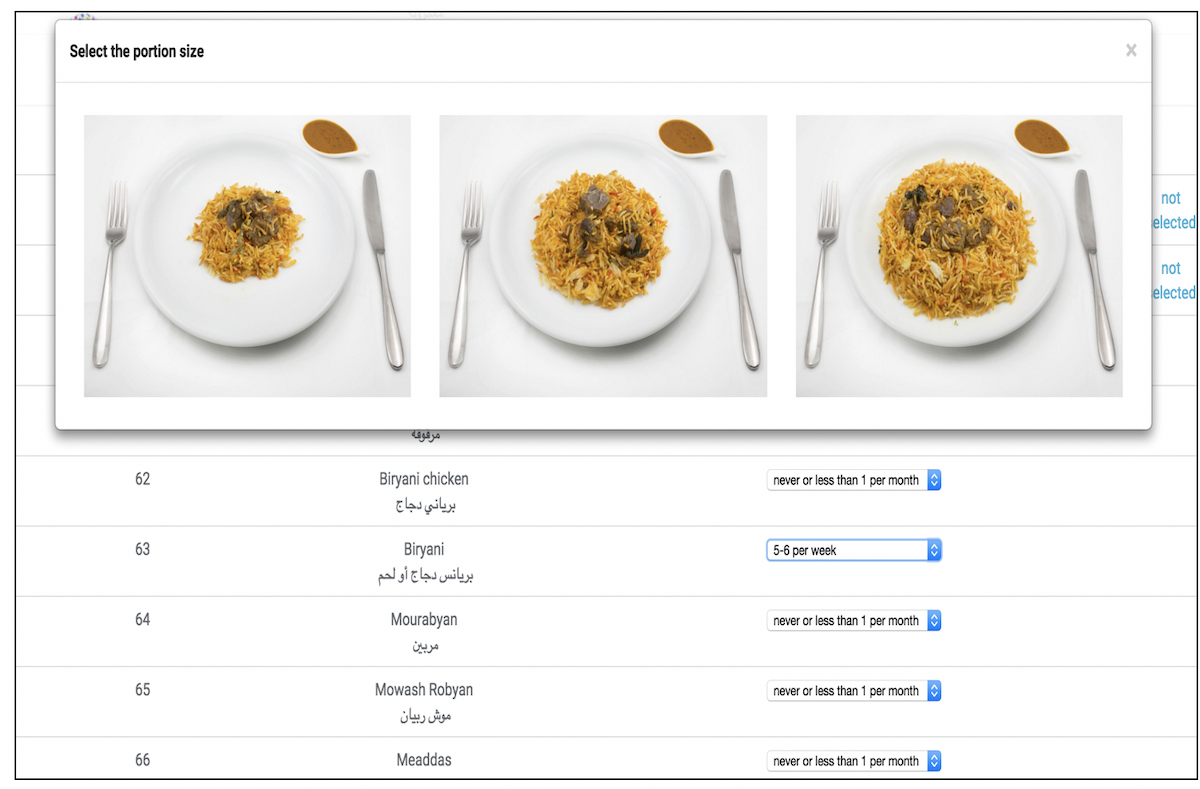
Screenshot of the web-based EatWellQ8 food frequency questionnaire illustrating the 3 portion size photographs for the assessment of the portion size.

The web-based EatWellQ8 semiquantitative FFQ was designed to measure the short-term nutritional and dietary intakes of adults in Kuwait. The design and development of the novel EatWellQ8 FFQ was led by researchers from the Hugh Sinclair Unit of Human Nutrition and the Biomedical Engineering section at the University of Reading. The validated Food4Me FFQ, the well-validated EPIC-Norfolk FFQ (version CAMB/PQ/6/1205), and a valid semiquantitative FFQ for Kuwait were used as a guide in the development of the novel FFQ to identify food items and categorize food into different food groups [13,14]. To ensure that the EatWellQ8 FFQ was suitable for use among people in Kuwait, participants were able to choose between 2 languages—Arabic and English. The novel FFQ comprised 146 food items (Multimedia Appendix 1) that represented food items and composite dishes commonly consumed in Kuwait. Several new foods that are commonly consumed in Kuwait were added to the existing food categories, for example, pomegranate, guava, and mango were added to the fruit list and *Lebanese bread and Iranian bread* were added to the bread and savory biscuit list. A new food section titled *Kuwaiti composite dishes* was added, which included 23 food items such as *Machboos Laham, Biryani, and Harees* to ensure that commonly consumed foods were included in the FFQ. In addition, traditional Kuwaiti desserts such as *Konafa, maamoul, and luqaimat* were added to the sweets and snacks section to ensure the inclusion of most of the commonly consumed food items. Alcoholic drinks and pork were removed from the FFQ as they were not commonly consumed items and also to respect the religious culture in Kuwait. Food items on the web-based FFQ appeared as a list where all the food items are displayed on a single page, as compared with displaying foods in food groups that are presented over several consecutive pages (Figure 1). 

### Photographs

Portion size photographs for 64 of the foods were derived from the Food4Me food portion size photograph list [15]. The remaining 83 food items were purchased from local supermarkets and local restaurants and bakeries in Kuwait. All foods were prepared and photographed at the DDI, Kuwait, over a period of 7 days or sessions by a professional photographer from DDI. Photographs were taken in the demo kitchen at DDI using the same lighting and a standard dining set of plates and cutlery that were positioned consistently for each session. All foods were weighed using calibrated portable food scales (Salter), and the calculated Food4Me portion sizes were used as a guide for all food items.

### The Four-Day WFR

Participants were asked to record all food items and beverages consumed over a four-day period that included 3 weekdays (Sunday to Thursday) and 1 weekend day (Friday to Saturday). Before beginning the WFR, participants were asked to attend a preliminary training session at DDI given by a dietitian on how to describe food products and use the provided food scales (Salter Disc Electronic Kitchen Scales SKU1036 WHSSDR). Participants were given the flexibility of estimating portion sizes when they were unable to weigh the food items (eg, when dining out).

### The Kuwaiti Validated FFQ

A total of 50 participants were asked to complete a PFFQ after completing the initial web-based FFQ. The Kuwait Validated FFQ is a self-administered semiquantitative FFQ that was developed in 2009. The FFQ was developed to target the frequency of consumption and portion sizes of food and beverages regularly consumed by the Kuwaiti population [14]. Standardized portions of the food items and beverages were used to estimate portion sizes, and 9 frequencies ranging from *never or once a month* to *more than 6 times/day* were used for frequency estimation [14]. The FFQ included questions on the average intake of 201 food items over the past 4 months. However, the time frame was reduced to 1 month for the purpose of the validation study. The food items were divided into the following 14 groups: *cereals*, *composite dishes*, *marag (stew)*, *soups*, *meat dishes*, *snacks*, *desserts*, *dairy products*, *beverages*, *fruits*, *vegetables*, *stuffed vegetables*, *salads*, and *miscellaneous*. The food intake (g/day) was calculated by multiplying the portion of each food listed in the FFQ by the frequency of consumption and by the nutrient composition of the food using the United States Department of Agriculture nutrient database [14].

### Dietary Intake Analysis

Estimated dietary intake data from the EatWellQ8 FFQ were generated automatically by the web-based EatWellQ8 app, which was described previously by Franco et al [17]. Nutritional composition and portion sizes of the 146 food items were calculated using the Food4Me food list [18], fifth and sixth editions of *The Composition of Foods* by McCance and Widdowson [19,20], the Kingdom of Bahrain Food Composition Tables [21], and the National Kuwait Food Composition List [22]. From these lists, the most commonly consumed food items were selected and used to calculate the composition of the lists of foods in the EatWellQ8 FFQ. The nutritional compositions of all the Kuwaiti composite dishes were determined using the Kingdom of Bahrain food composition list and a Kuwaiti food composition list [21,22]. Portion sizes were primarily derived using the Food4Me food list [15,16]. To calculate the portion sizes, the food codes for each of the frequently consumed foods were identified from the Food4Me database and used to formulate the code for the food items in the FFQ. PASW Statistics version 24 (SPSS Inc) was used to calculate the 25th, 50th, and 75th percentile of daily food intake, which corresponds, respectively, to small, medium, and large portion of these foods when consumed by the general population [15]. Estimated nutrient intakes for the PFFQ were analyzed using a Microsoft Excel file that was based on the web-based EatWellQ8 programmed system. The four-day WFR intakes were analyzed using Nutritics software (version 1.8, database MW6, Nutritics Ltd, Co).

### Over-Underreporting

Participants’ results were excluded from the analysis if their daily energy intake was found to be less than 500 kcal or greater than 4500 kcal in any of the methods [23].

### Statistical Analysis

Statistical analyses were performed using SPSS (version 24.0, PASW). Normality was assessed using the Shapiro-Wilk test, and log transformation was used for nonparametric data when necessary. A paired two- tailed *t* test was performed to assess differences in participants’ energy intake (kcal) between the methods used. SDs and mean nutrient intakes were calculated for baseline, repeated EatWellQ8 FFQ, PFFQ, and four-day WFR. Comparisons between nutrient intakes were performed using a general linear model analysis, which was controlled further for energy and gender, where there was a significant interaction between nutrient intake and gender. To check for normality, data were analyzed using a Shapiro-Wilk test, and either the Pearson or the Spearman correlation coefficient (SCC) was used for normally or nonnormally distributed data, respectively. Correlations were considered statistically significant if the *P* value was <.05.

To test for agreement between the different dietary intake methods and repeated EatWellQ8 FFQ, cross-classification of nutrient intakes to assess the percentage of participants classified into the following quartiles: exact agreement (percentage of cases cross-classified into the same quartile), exact agreement plus adjacent (percentage of cases cross-classified into the same or adjacent quartile), disagreement (percentage of cases cross-classified 2 quartiles apart), and extreme disagreement (percentage of cases cross-classified into extreme quartiles). The Bland-Altman [24] method was used to further analyze the LOA for energy intakes and macronutrients between the repeated EatWellQ8 FFQ and between the 3 methods (EatWellQ8 FFQ, WFR, and PFFQ). On the basis of the Bland-Altman method, dietary intake methods were found to be repeatable or comparable if greater than 95% of the data plots fell within the 2 SD of the mean (LOA) and by calculating the bias calculated by the mean difference and SD of the differences.

## Results

### Overview

**Figure 2:**
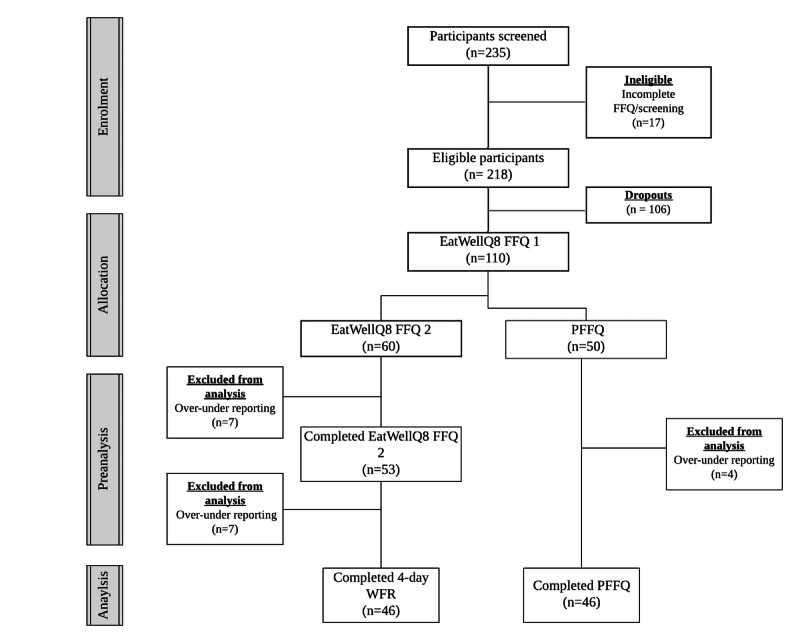
Participant flow during the study.

Of the 235 participants screened for the study, 218 were found to be eligible. Participants were excluded (n=17) because of incomplete FFQs or not fulfilling the screening requirements because of medication use, food allergies, or an existing illness. The mean BMI, weight, and height of participants included in the study were 25.6 kg/m^2^ (SD 4.4), 70.3 kg (SD 14.0), and 165.5 cm (SD 8.6), respectively, and the mean BMI, weight, and height of dropouts were 25.7 kg/m^2^ (SD 4.3), 70.7 kg (SD 13.8), and 166 cm (SD 8.2), respectively. A high dropout rate of 48.6% (106/218) was found, which was mainly because of participants’ unwillingness to complete all aspects of the study. The mean completion time of the EWQ8 FFQ was 14.3 minutes (95% CI 12.9-15.3) [17]. A total of 110 participants completed the EatWellQ8 FFQ1, of which 60 completed EatWellQ8 FFQ2 and a four-day WFR and 50 were asked to complete a PFFQ. In total, 18 participants were excluded from the analysis because of reported energy intakes of <500 kcal or >4500 kcal [23]. Removal of under-overreporters did not have an impact on the outcomes of reproducibility and validity (data not shown). Of these, 53 participants completed the second EatWellQ8 FFQ, 46 completed EatWellQ8 FFQ2 and the four-day WFR, and 46 participants completed the PFFQ. An illustration of the flow of the participants is shown in Figure 2. Demographic characteristics based on self-report are shown in Table 1. No significant differences were found between age and BMI for females and males. A higher percentage of females completed both studies (60/92, 65% in the validation study and 35/53, 66% in the reproducibility study).

**Table 1:** Demographic characteristics of participants who completed the reproducibility and validation studies.

Study		Population, n (%)	Demographic characteristics, mean (SD)			
			Age (years)	Weight (kg)	Height (cm)	BMI (kg/m^2^)
**Reproducibility**						
	All	53 (100)	37 (9.9)	70.3 (13.9)	165.5 (8.6)	25.6 (4.4)
	Female	35 (66)	36 (9.8)	64.9 (11.1)	161.6 (6.2)	24.9 (4.6)
	Male	18 (34)	39 (9.7)	80.8 (12.8)	173.3 (7.3)	26.8 (3.5)
**Validation**						
	All	92 (100)	36 (8.3)	71.9 (15.5)	167.4 (8.1)	25.2 (4.4)
	Female	60 (65)	37 (9.2)	65.6 (11.9)	163.8 (5.6)	24.3 (4.4)
	Male	32 (35)	34 (6.1)	82.6 (15.4)	173.6 (7.9)	27.0 (3.9)

### Reproducibility of the EatWellQ8 FFQ

#### Comparison of Nutrient Intake Between Repeated EatWellQ8 FFQs

No significant differences were found between macronutrient and micronutrient intake evaluated in FFQ1 and FFQ2 (Table 2). Correlations were found to be significant for all nutrients (*P*=.01) and ranged from 0.37 (polyunsaturated fatty acids [FAs], percentage total energy [%TE]) to 0.82 (iron), with a mean value of *r*=0.67 (SD 0.14; 95% CI 0.11-0.89; Multimedia Appendix 2 Table S1). Adjustments for energy and gender did not modify these correlations. Results of the cross-classifications for percentage of participants classified into quartiles of exact agreement ranged from 40% (polyunsaturated FAs, %TE) to 62% (total folate). Classifications of exact agreement plus adjacent regions ranged from 77% (monounsaturated FAs, %TE) to 100% (energy, kcal). Disagreement was relatively low, the mean percentage of participants classified into quartiles of disagreement was 8%, and the mean of participants classified as having extreme disagreement was 1.40%.

**Figure 3:**
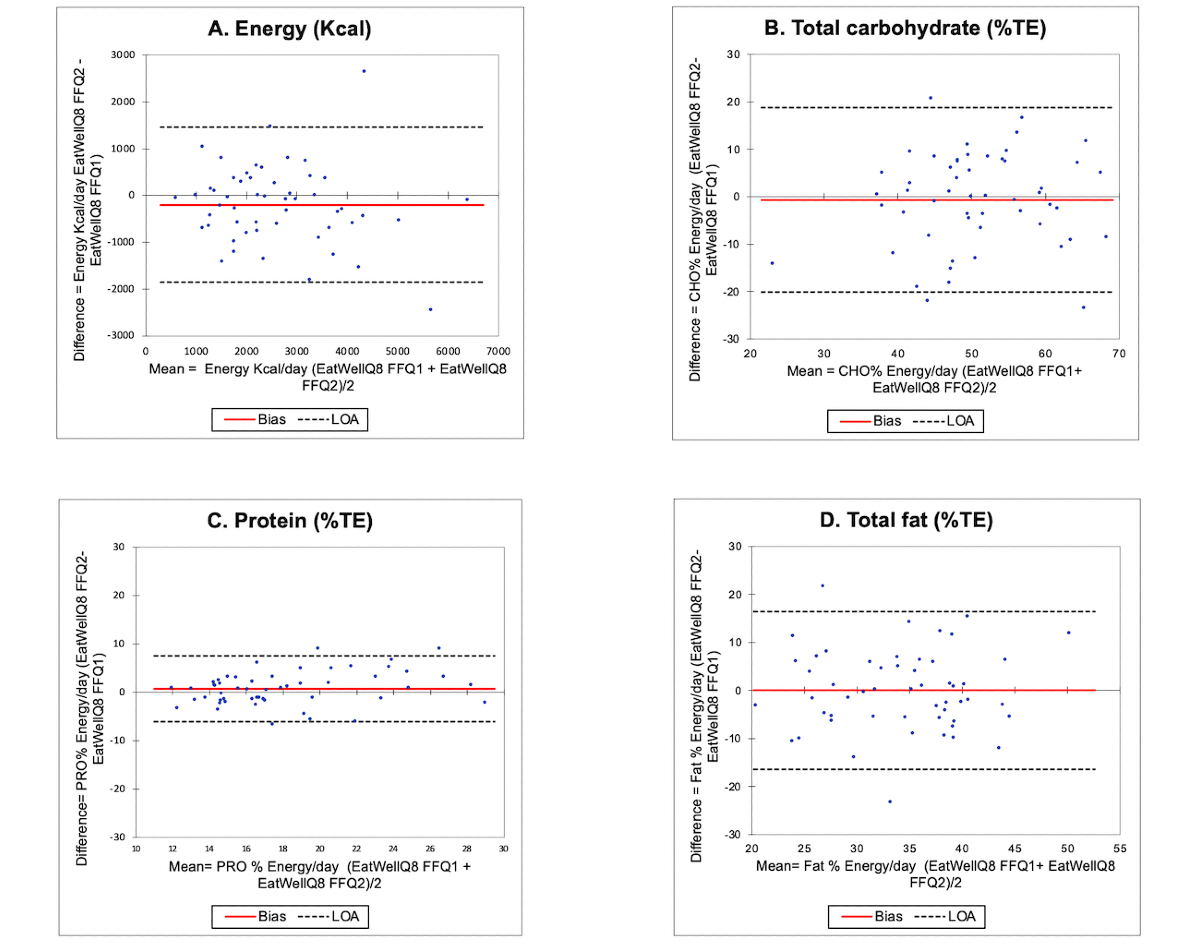
Reproducibility study of Bland-Altman plots for (a) energy, (b) total carbohydrate, (c) protein, and (d) total fat with the bias (mean difference) and limits of agreement. The solid line represents the bias (mean difference), and the dotted lines represent the limits of agreement. %TE: percentage total energy; CHO: carbohydrates; PRO: protein.

The Bland-Altman plots for estimates of energy (kcal), protein (%TE), total fat (%TE), and carbohydrate (%TE) intakes are shown in Figure 3. Good agreement was found in the Bland-Altman plots, as the majority of the cases fell within the 95% LOA. The EatWellQ8 FFQ presented good reproducibility for the evaluation of daily fat intake, with less than 4% of cases outside the LOA. For energy and carbohydrate, less than 6% fell outside the LOA and 7% for protein. On the basis of the LOA values, greater agreement was found for protein (%TE) compared with energy, total fat, and total carbohydrate (%TE). No significant bias was identified for any nutrient. Variation between estimates of energy and energy-adjusted macronutrient intakes increased with higher mean intakes (Figure 3).

**Table 2:** Mean daily energy and nutrient intakes estimated by repeated measures of the web-based EatWellQ8 food frequency questionnaire (N=53).

Nutrient	EatWellQ8 FFQ1^a^, mean (SD)	EatWellQ8 FFQ2, mean (SD)	*P* value^b^	*P* value^c^
Energy (kcal)	2724 (1355)	2524 (1232)	.09^d^	N/A^e^
Total fat (g)	104.7 (56.2)	96.1 (50.2)	.79	.79
Total fat (%TE^f^)	34.2 (7.7)	34.2 (7.8)	.96	.96
SFA^g^ (g)	43.5 (27.0)	38.5 (22.1)	.49	.49
SFA (%TE)	14.0 (4.4)	13.6 (4.8)	.73	.73
MUFA^h^ (g)	45.4 (25.0)	40.0 (21.4)	.34	.34
MUFA (%TE)	14.9 (4.2)	14.3 (3.9)	.41	.41
PUFA^i^ (g)	18.0 (9.1)	17.5 (8.9)	.46	.46
PUFA (%TE)	6.1 (1.7)	6.4 (1.7)	.58	.58
Omega 3 (g)	0.17 (0.2)	0.20 (0.4)	.47	.47
Protein (g)	117.8 (57.3)	111.4 (52.2)	.84	.84
Protein (%TE)	17.7 (4.1)	18.5 (5.1)	.53	.54
Carbohydrate (g)	348 (197)	323 (182)	.87	.88
Carbohydrate (%TE)	51.1 (9.8)	50.3 (10.7)	.76	.77
Total sugars (g)	149 (91)	134 (79)	.69	.69
Total sugars (%TE)	22.1 (9.2)	21.4 (7.3)	.67	.67
Calcium (mg)	1288 (682)	1192 (633)	.86	.86
Total folate (µg)	405 (204)	358 (167)	.29	.29
Iron (mg)	16.8 (9.5)	15.0 (7.8)	.41	.41
Total carotene (µg)	6581(6161)	5548 (4079)	.41	.41
Riboflavin (mg)	2.3 (1.2)	2.2 (1.1)	.90	.89
Thiamin (mg)	2.1 (1.1)	1.9 (0.9)	.78	.77
Vitamin B6 (mg)	2.8 (1.2)	2.7 (1.2)	.85	.85
Vitamin B12 (µg)	5.2 (3.3)	5.4 (3.4)	.33	.33
Vitamin C (mg)	200 (116)	183 (135)	.77	.76
Vitamin A RE^j^ (µg)	1319 (1048)	1153 (718)	.48	.48
Retinol (µg)	241 (190)	258 (191)	.15	.15
Vitamin D (µg)	3.1 (2.3)	3.4 (3.2)	.36	.36
Vitamin E (mg)	15.4 (7.5)	14.3 (7.6)	.89	.89
Sodium (mg)	3159 (1570)	2948 (1485)	.99	.99

^a^FFQ: food frequency questionnaire.

^b^Controlled for energy.

^c^Controlled for energy and gender.

^d^Value derived from paired sample *t* test.

^e^N/A: not applicable.

^f^%TE: percentage total energy.

^g^SFA: saturated fatty acid.

^h^MUFA: monounsaturated fatty acid.

^i^PUFA: polyunsaturated fatty acid.

^j^RE: retinol equivalent.

#### Comparison of Food Group Intakes Between Repeated EatWellQ8 FFQ

Food items were categorized into 32 food groups to assess the differences between repeated administrations of the web-based EatWellQ8 FFQ. SCCs ranged from 0.40 (savories) to 0.93 (meat products) with a mean value of *r*=0.67 (SD 0.14; 95% CI 0.11-0.95; Multimedia Appendix 2 Table S2). Significant correlations were found for all food groups (*P*=.01). The cross-classification of participants classified into quartiles of exact agreement ranged from 45% (salad vegetables) to 76% (meat products). Moderately high classifications of exact agreement and adjacent were found, which ranged from 66% (confectionary and savory snacks) to 98% (meat products).

### Validation of the EatWellQ8 FFQ

#### Comparison of Nutrient Intakes Between the EatWellQ8 FFQ and the Kuwaiti PFFQ

No significant differences were found between 70% of the macronutrients and micronutrients evaluated by the EatWellQ8 FFQ1 and PFFQ (Table 3). Estimated energy intakes were found to be significantly higher (difference 398 kcal/day) and 17% higher (*P*<.001) in the EatWellQ8 FFQ1 than in the PFFQ.

After controlling for energy, similar estimated intakes of macronutrients and micronutrients were observed for EatWellQ8 FFQ1 and the PFFQ except for saturated fatty acids (SFAs) and monounsaturated fatty acids (MUFAs; g, %TE), which were significantly higher for EatWellQ8 FFQ than for PFFQ (*P*<.001). Furthermore, the estimated intakes of total folate (*P*=.01), retinol (*P*<.001), and vitamin B12 (*P*<.001) were higher in the PFFQ than in the EatWellQ8 FFQ.

With the exception of omega 3 FAs and retinol, correlations were found to be significant for all nutrients (*P=*.01) and ranged from 0.42 (vitamin D) to 0.73 (energy), with a mean value of *r*=0.54 (SD 0.12; Multimedia Appendix 2 Table S3). However, large variations were found in 95% CI ranging from −0.02 to 0.84, and weak 95% CIs were found for retinol (−0.02) and omega 3 FAs (−0.11). The results of the cross-classifications for percentage of participants classified into quartiles of exact agreement ranged from 35% (total fat) to 57% sodium (Na), exact agreement plus adjacent, ranging from 76% (total fat, %TE) to 93% (energy) with low levels in disagreement (13.41%) and extreme disagreement (2.40%).

**Table 3:** Mean daily energy and nutrient intakes estimated by the web-based EatWellQ8 food frequency questionnaire and a paper-based food frequency questionnaire and general linear model results (N=46).

Nutrient	EatWellQ8 FFQ^a^, mean (SD)	PFFQ^b^, mean (SD)	*P* value^c^	*P* value^d^
Energy (kcal)	2297 (779)	1899 (505)	<.001^e^	N/A^f^
Total fat (g)	92.1 (40.8)	69.1 (23.4)	.12	.12
Total fat (%TE^g^)	35.5 (7.8)	32.4 (5.1)	.13	.13
SFA^h^ (g)	38.4 (17.9)	26.9 (9.1)	.01	.01
SFA (%TE)	14.9 (4.8)	12.6 (2.7)	.01	.01
MUFA^i^ (g)	39.5 (19.5)	26.4 (9.7)	<.001	.001
MUFA (%TE)	15.1 (4.3)	12.3 (2.3)	<.001	.001
PUFA^j^ (g)	16.1 (7.8)	13.1 (6.5)	.64	.58
PUFA (%TE)	6.2 (1.7)	6.1 (1.8)	.93	.96
Omega 3 (g)	0.1 (0.2)	0.3 (0.3)	<.001	<.001
Protein (g)	104 (41)	93 (41)	.35	.33
Protein (%TE)	18.2 (5.0)	19.4(6.1)	.39	.37
Carbohydrate (g)	280 (102)	241 (70)	.53	.53
Carbohydrate (%TE)	49.1 (10.5)	51.2 (7.9)	.53	.54
Total sugars (g)	125 (52)	105 (32)	.56	.53
Total sugars (%TE)	22.3 (8.8)	22.6 (6.2)	.53	.53
Calcium (mg)	1126 (542)	933 (358)	.86	.84
Total folate (µg)	323 (135)	328 (116)	.01	.01
Iron (mg)	13.5 (5.7)	11.5 (4.0)	.48	.49
Total carotene (µg)	5042 (3430)	4781 (4325)	.82	.82
Riboflavin (mg)	2.0 (0.9)	1.8 (0.7)	.30	.31
Thiamin (mg)	1.7 (0.7)	1.5 (0.4)	.18	.18
Vitamin B6 (mg)	2.4 (0.8)	2.1 (0.8)	.77	.77
Vitamin B12 (µg)	4.8 (2.8)	5.5 (3.2)	<.001	<.001
Vitamin C (mg)	163 (141)	156 (98)	.67	.68
Vitamin A RE^k^ (µg)	1054 (590)	1110 (796)	.22	.22
Retinol (µg)	237 (138)	387 (452)	<.001	<.001
Vitamin D (µg)	2.6 (2.0)	2.3 (1.7)	.48	.45
Vitamin E (mg)	12.7 (7.0)	9.5 (3.9)	.44	.45
Sodium (mg)	2701 (1058)	2102 (771)	.21	.21

^a^FFQ: food frequency questionnaire.

^b^PFFQ: paper-based food frequency questionnaire.

^c^Controlled for energy.

^d^Controlled for energy and gender.

^e^Value derived from paired sample *t* test.

^f^N/A: not applicable.

^g^%TE: percentage total energy.

^h^SFA: saturated fatty acid.

^i^MUFA: monounsaturated fatty acid.

^j^PUFA: polyunsaturated fatty acid.

^k^RE: retinol equivalent.

**Figure 4:**
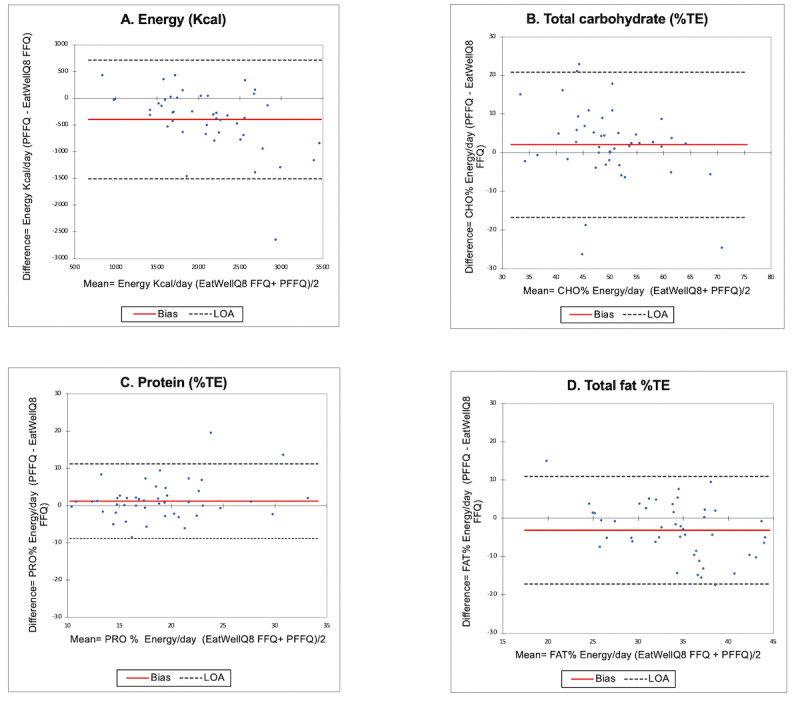
Validation study of Bland-Altman plots comparing the EatWellQ8 food frequency questionnaire (to a paper-based food frequency questionnaire for (a) energy, (b) total carbohydrate, (c) protein, and (d) total fat with the bias (mean difference) and limits of agreement. The solid line represents the bias (mean difference), and the dotted lines represent the limits of agreement. %TE: percentage total energy; CHO: carbohydrates; FFQ: food frequency questionnaire; LOA: limit of agreement; PRO: protein; PFFQ: paper-based food frequency questionnaire.

Overall, moderate agreement was found in the Bland-Altman plots between the EatWellQ8 and the paper form of PFFQ, with 87% of all cases falling within the 95% LOA (Figure 4). The EatWellQ8 FFQ presented good validation for the evaluation of energy and daily intake of fat, with approximately 4% of cases falling outside the LOA. For daily intake of carbohydrates, less than 6% fell outside the LOA and, for protein, 8% fell out of the LOA. Protein (%TE) had the narrowest LOA, which signifies better agreement compared with energy, total fat (%TE), and carbohydrate (%TE). The bias (mean difference) between energy intakes was significantly higher (398 kcal/day), with greater intakes reported in the EatWellQ8 FFQ. A high mean bias was found for total fat (−3.05%TE) compared with total carbohydrate (2.67%TE) and protein (1.20 %TE). No other significant differences were observed.

#### Comparison of Food Group Intakes Between the EatWellQ8 FFQ and the Kuwaiti PFFQ

SCCs ranged from 0.51 (bananas) to 0.22 (fish and fish product or dishes; 95% CI −0.07 to 0.71; Multimedia Appendix 2 Table S4). With the exception of fish and fish products or dishes, significant correlations were found for all food groups (*P*=.047). The cross-classification percentages of participants classified into quartiles of exact agreement ranged from 60% (soups, sauces, and miscellaneous foods) to 24% (white bread). Classifications of exact agreement plus adjacent ranged from 65% (ice cream, creams, and desserts) to 82% (teas and coffees). The mean percentage of participants classified into quartiles of disagreement was 15% and, for extreme disagreement, it was 9%.

#### Comparison of Nutrient Intakes Between the EatWellQ8 FFQ and a Four-Day WFR

Estimated macronutrient intakes were found to be similar between the EatWellQ8 FFQ and the 4-day WFR after controlling for energy (Table 4). However, estimated intakes of SFA (*P*=.001), total carbohydrates (*P*=.03), and total sugars (g, %TE; *P*=.03) were significantly higher in the EatWellQ8 FFQ than in the four-day WFR. Significantly higher estimated intakes of omega 3 FAs, folate, total carotene, thiamin, vitamin B6, vitamin C, vitamin A retinol equivalent, and Sodium (*P=*.02) were found for the EatWellQ8 FFQ compared with the four-day WFR. Similar results were found after controlling for both energy and gender.

Significant correlation for all nutrients was found at the *P*=.01 level, correlation coefficients ranged from 0.40 (iron) to 0.88 (energy), with a mean value of *r*=0.61 (SD 0.13; 95% CI 0.13-0.93; Multimedia Appendix 2 Table S5). The percentage of volunteers classified into quartiles of exact agreement ranged from 28% (polyunsaturated fatty acid, g) to 67% (energy, kcal). Values were higher for classifications of exact agreement plus adjacent and ranged from 71% (MUFA, %TE) to 95% (protein, g). The mean percentage of volunteers classified into quartiles of disagreement was 11%, and less than 2% of volunteers were classified as having extreme disagreement.

**Figure 5:**
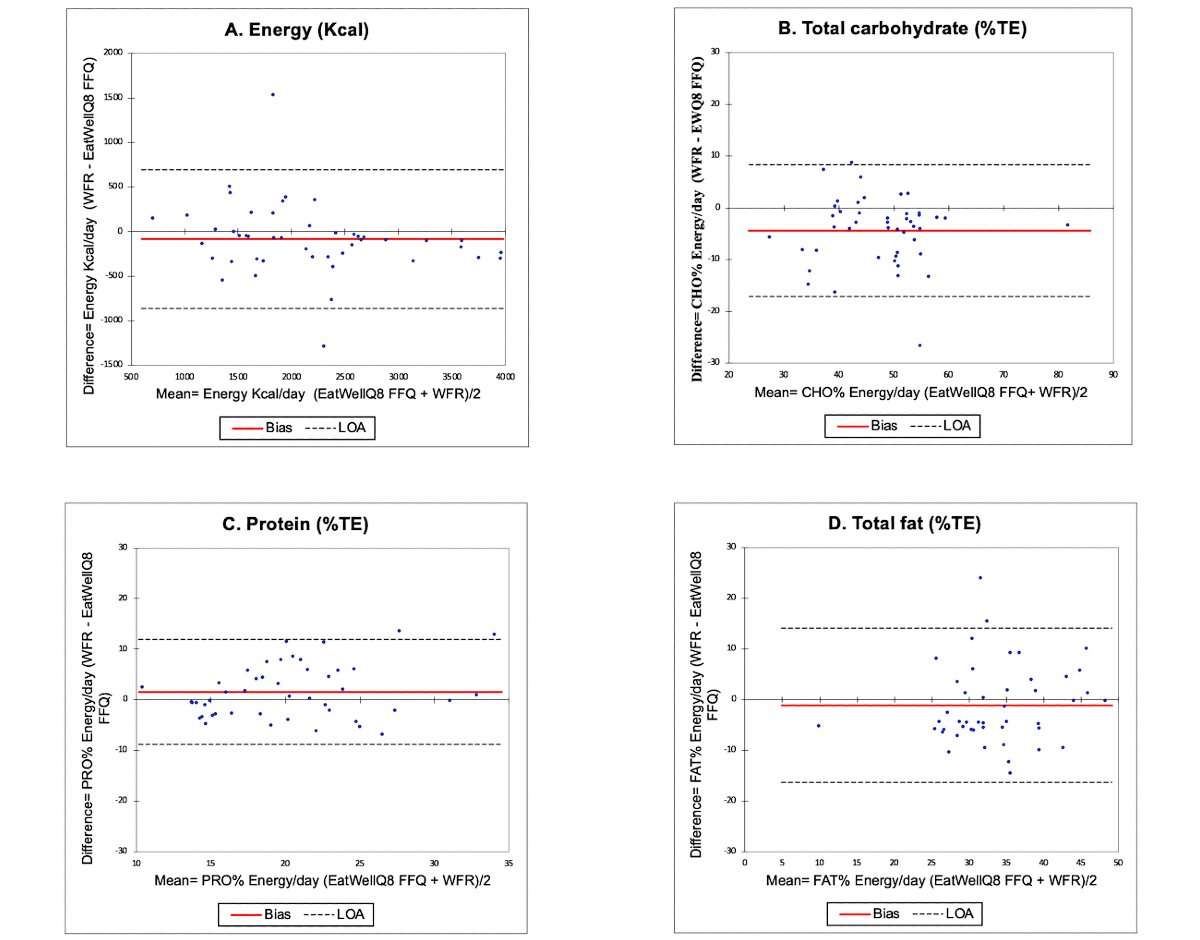
Validation study of Bland-Altman plots comparing the EatWellQ8 food frequency questionnaire with a four-day weighed food record for (a) energy, (b) total carbohydrate, (c) protein, and (d) total fat with the bias (mean difference) and limits of agreement. The solid line represents the bias (mean difference), and the dotted lines represent the limits of agreement. %TE: percentage total energy; CHO: carbohydrates; FFQ: food frequency questionnaire; PRO: protein; WFR: weighed food record.

In total, good agreement between the methods was found, with less than 5% of cases falling outside of the LOA for all of the plots (Figure 5). On the basis of the LOA values, the highest agreement was found for protein (%TE) compared with energy total carbohydrate (%TE) and total fat (%TE). Bias (mean difference) between energy intakes was small (81 kcal/day), with greater values estimated in the EatWellQ8 FFQ. Higher bias for energy-adjusted total carbohydrate (4.39%TE) and total fat (1.20%TE) intake was measured in the EatWellQ8 FFQ. However, a higher bias for energy-adjusted protein (1.65%TE) intakes was measured in the four-day WFR.

**Table 4:** Mean daily energy and nutrient intakes estimated by the web-based EatWellQ8 food frequency questionnaire and a four-day weighed food record and general linear model results (N=46).

Nutrient	EatWellQ8 FFQ^a^, mean (SD)	WFR^b^ four-day, mean (SD)	*P* value^c^	*P* value^d^
Energy (kcal)	2199 (862)	2119 (772)	.17^e^	N/A^f^
Total fat (g)	84.2 (39.0)	74.3 (27.6)	.08	.08
Total fat (%TE^g^)	34.0 (7.4)	32.8 (8.5)	.47	.48
SFA^h^ (g)	36.8 (18.2)	28.0 (12.0)	.001	.001
SFA (%TE)	14.8 (3.8)	11.9 (2.8)	.002	.002
MUFA^i^ (g)	35.8 (17.9)	29.8 (13.9)	.04	.04
MUFA (%TE)	14.5 (4.1)	13.2 (5.1)	.17	.17
PUFA^j^ (g)	14.5 (6.8)	11.7 (6.5)	.03	.03
PUFA (%TE)	5.9 (1.6)	5.2 (2.7)	.07	.07
Omega 3 (g)	0.1 (0.2)	0.3 (0.3)	.002	.002
Protein (g)	106 (50)	112 (55)	.11	.11
Protein (%TE)	19.3 (5.2)	21.00 (6.44)	.16	.16
Carbohydrate (g)	272 (117)	238 (102)	.03	.03
Carbohydrate (%TE)	49.8 (9.7)	45.4 (9.5)	.03	.03
Total sugars (g)	130 (74)	104 (63)	.03	.03
Total sugars (%TE)	23.1 (8.2)	19.3 (8.1)	.03	.02
Calcium (mg)	1191 (668)	1005 (508)	.07	.07
Total folate (µg)	345 (152)	288 (121)	.02	.02
Iron (mg)	13.4 (5.4)	11.9 (5.0)	.13	.13
Total carotene (µg)	5106 (4439)	3407 (3480)	.04	.04
Riboflavin (mg)	2.1 (1.2)	1.9 (1.1)	.40	.40
Thiamin (mg)	1.8 (0.8)	1.4 (0.5)	.001	.001
Vitamin B6 (mg)	2.5 (1.2)	2.0 (0.9)	.01	.01
Vitamin B12 (µg)	5.3 (3.5)	6.6 (11.3)	.37	.38
Vitamin C (mg)	178 (153)	126 (80)	.04	.04
Vitamin A RE^k^ (µg)	1057 (766)	739 (497)	.02	.02
Retinol (µg)	223 (147)	202 (112)	.56	.57
Vitamin D (µg)	2.6 (1.9)	2.9 (1.8)	.26	.26
Vitamin E (mg)	11.8 (6.7)	10.6 (5.3)	.39	.39
Sodium (mg)	2552 (898)	2010 (815)	.001	.001

^a^FFQ: food frequency questionnaire.

^b^WFR: weighed food record.

^c^Controlled for energy.

^d^Controlled for energy and gender.

^e^Value derived from paired sample *t* test.

^f^N/A: not applicable.

^g^%TE: percentage total energy.

^h^SFA: saturated fatty acid.

^i^MUFA: monounsaturated fatty acid.

^j^PUFA: polyunsaturated fatty acid.

^l^RE: retinol equivalent.

#### Comparison of Food Group Intakes Between the EatWellQ8 FFQ and the 4-Day WFR

The correlation coefficients and cross-classifications of mean food group intakes between the EatWellQ8 FFQ and the four-day WFR. SCCs ranged from 0.30 (bananas) to 0.88 (red meat; Multimedia Appendix 2 Table S6). Significant correlations were found for all food groups (95% CI 0.01-0.91), and weak 95% CIs were found for breakfast cereals and porridge (0.08) and bananas (0.01; *P*=.046). The cross-classification percentages of participants classified into quartiles of exact agreement ranged from 28% (green vegetables) to 65% (wholegrain and brown breads and rolls). Relatively high classifications of exact agreement plus adjacent were found, ranging from 71% (green vegetables) to 97% (red meat). The mean percentage of participants classified into quartiles of disagreement was 11% and, for extreme disagreement, it was 5%.

## Discussion

### Principal Findings

This study aimed to evaluate the reproducibility of the EatWellQ8 FFQ and to test its relative validity against a semiquantitative Kuwaiti PFFQ and a four-day WFR. The EatWellQ8 FFQ has been developed to assess dietary and nutrient intakes in the EatWellQ8 study to investigate the effectiveness of delivering personalized face-to-face dietary advice compared with web-based dietary advice in Kuwait. It included images of 3 different portion sizes for each food item to aid in portion size estimation and food recognition. The need to develop a culturally sensitive FFQ that reflected the diet of the Kuwaiti population was necessary to avoid misclassifications of dietary intakes. The results of this study indicated that the EatWellQ8 FFQ is a suitable tool with moderate validity for the assessment of nutrient and food intake in a sample of healthy adults living in Kuwait.

### Reproducibility

Overall, the EatWellQ8 FFQ had good reproducibility for the estimation of nutrient intake and food groups over a period of 4 weeks. The correlation coefficients for all nutrients were significant, compared with previous studies, and nearly all fell within the acceptable range of 0.5 to 0.7 for reproducibility trials proposed by Cade et al [5,16,25-29]. Similarly, strong associations were found between food groups with a mean SCC value of 0.67, which was comparable with previous web-based FFQ reproducibility studies by Fallaize et al [16] and Vereecken et al [30] that reported mean correlations of 0.75 and 0.64, respectively. However, a limitation in the trial by Vereecken et al [30] was the short assessment time between repeatability of the FFQs of only 1 to 2 weeks, which may have impacted the power of the trial. The usage of correlation analysis to assess agreement has been questioned as it only measures the degree of association between 2 variables and does not assess agreement [5,24]. Cross-classifications into quartiles of agreements and Bland-Altman plots were used to measure agreement. Analysis of cross-classifications of exact plus adjacent agreement of energy, nutrients, and food group intakes (mean value of 88%) indicated a high level of agreement and a low level of misclassification (<10%), similar to the results of previous web-based FFQ studies [10,16]. The high level of reproducibility may be in part due to the short period (4 weeks) between FFQ administrations, as true changes in dietary intakes are less likely to occur within a short period [31]. These data were also supported by the level of reproducibility from the Bland-Altman analysis for energy-controlled total protein, fat, and carbohydrate, which compared with findings from Fallaize et al [16] and Papazian et al [32]. Limitations to the trial by Papazian et al [32] were the relatively small sample size of 38 and the short interval time between FFQ administrations of 3 weeks, which may have impacted trial outcomes.

Results from several previous reproducibility studies have shown greater intakes in energy and nutrient intakes in the first FFQ compared with the second FFQ [10,16,26,29,33]. No significant differences between intakes were observed in this study, except for SFA and MUFA; however, quantitatively higher estimated energy and nutrient intakes were found in the initial administration of the EatWellQ8 FFQ compared with the second administration, which may be because of questionnaire boredom as a result of the short period between FFQs [5,34]. However, it has been proposed that the good reproducibility of the EatWellQ8 FFQ may be influenced by the short interval between FFQ administrations. It has been proposed that if the interval time between FFQs is short (1-6 months), participants’ memory may influence the outcome, leading to overestimation in the reproducibility [16,35]. In contrast, underestimation was found in FFQs with longer time intervals (>6 months) because of changes in dietary habits [36]. We were keen for participants not to change dietary habits and explicitly asked for no change, which could have contributed to good reported reproducibility in our study. An additional factor that may have contributed to the good reproducibility is the use of photographs as an aid to food portion size estimation. It has been proposed that reproducibility is enhanced in FFQs that take into account food portion sizes, especially when participants are allowed to specify their own portion size [5].

### Relative Validity

Overall, the results of the validation study demonstrated moderate to weak agreement between the EatWellQ8 FFQ and 2 dietary collection tools for the estimation of energy and nutrient intakes: a PFFQ and a four-day WFR. This was reflected by the higher level of bias being estimated by the EatWellQ8 FFQ for macronutrients (except for protein) and the level of disagreement in the cross-classifications, particularly in relation to food groups. This was also reflected by the large variations in the 95% CI range for both nutrients and food groups. The mean absolute intakes for most of the nutrients did not differ significantly between the tools. However, significant differences were found for specific FA (eg, SFA), which could possibly be because of differences in the food items presented in the FFQs. Similar to previous findings by Forster et al [15] and Beasley et al [26], compared with a PFFQ, the EatWellQ8 FFQ estimates of energy intake were significantly higher (*P*<.001). It has been reported that underestimation of dietary intake is common in PFFQs, which has been proposed to be because of errors such as skipped questions and a broad or vague use of portion size description [28].

With the exception of 2 nutrients (omega 3 FA and retinol), SCC fell within the range considered acceptable for FFQ validation trials from 0.4 to 0.7 [37,38]. The mean SCC for nutrients attained in this trial (*r*=0.54) was higher than that reported for a web-based FFQ validated against a PFFQ (*r*=0.47) and the one reported in the validation of a web-based diet history questionnaire against a four-day WFR [26,39]. The weakest SCC was found for specific FA (eg, omega 3 FA), and this finding was supported further in the results of cross-classifications, which also showed the least agreement for FA. This may be explained by a higher within-subject variation in fat intake. In this study, correlation coefficients for food groups were found to be relatively lower than the correlations found in trials by Forster et al [15] and Boeckner et al [40] that ranged from 0.42 to 0.90, which may be because of differences in the length of the PFFQs and number of food groups analyzed. Wide variations were observed in SCC between the EatWellQ8 FFQ and the PFFQ for food groups, suggesting that participants were able to estimate certain food items (eg, bananas) more accurately [41]. The proposed reasons for these variations are answering fatigue as a result of the length of the FFQs and may be a result of an overestimation of items that are perceived as healthy, such as vegetables and fruits, which is also common in other web-based FFQs [16,39].

The results of cross-classification for energy and nutrient intakes indicated that most participants were classified into exact plus adjacent quartiles that ranged from 76% to 93% and extreme disagreement or misclassification was <5% for most nutrients. Comparable cross-classifications that ranged from 77% to 99% were found when the Food4Me web-based FFQ was validated against the well-validated EPIC-Norfolk FFQ [15]. However, disagreement was high for food groups, especially for the food groups that were located at the end of PFFQ (eg, ice cream, creams, and desserts), suggesting answering fatigue. The results of the Bland-Altman plots showed moderate agreement between the methods for estimates of energy and energy-adjusted macronutrient intakes, and the least agreement was for %TE of fat. A possible reason for the disagreement between the tools may be participants’ inability to assess portion sizes accurately using the PFFQ because of the lack of food photographs of portion sizes.

The EatWellQ8 FFQ was found to estimate higher energy, nutrient, and food group intakes compared with the 4-day WFR. These results were expected as it has been found in previous studies that FFQs that contain >100 items tend to show an overestimation in energy, nutrient, and food intake compared with WFR and 24-hour recalls, which may be because of underestimation of the latter methods or overestimation of FFQ [35,42]. Comparable percentages of individuals classified into quartiles of exact agreement (mean 49%) and exact plus adjacent agreement (mean 84%) were found between the EatWellQ8 FFQ and the four-day WFR for energy, nutrient intakes, and food groups, and low levels of disagreement were found. Cross-classifications were within the range reported in previous trials, which were both validated against WFRs [16,43]. The results of the Bland-Altman plots established good agreement between the 2 methods for energy and energy-adjusted macronutrient intakes. In addition, 28 of 30 nutrients measured had a correlation of higher than the 0.40 threshold recommended by Cade et al [5]. The relatively short period between administrations of the 2 methods (7 to 10 days) could have contributed to the high correlations. Highly variable SCCs were found for food group intakes that ranged from 0.29 for bananas to 0.88 for red meat, with a mean value of 0.55. Results from previous studies found similarly high variations that ranged from 0.09 to 0.95 [16,23,44,45]. It may be difficult to compare our results with previous studies because of differences in the type of food record used, food items included in specific food groups, and differences in the time intervals in each of the studies. Variations between the EatWellQ8 FFQ and the 4-day WFR were greatest for bananas, green vegetables, meat products, and tinned fruit or vegetables. This may be because of overestimations by the FFQ of foods perceived as healthy and can be because of the relatively short WFR (4 days), which may not reflect the individuals’ dietary habits compared with the EatWellQ8 FFQ, which conveys the diet over the previous month, especially for foods that are not consumed regularly [46]. The wide variations observed in correlations between the EatWellQ8 FFQ and the four-day WFR may indicate whether volunteers could accurately estimate the consumption of some food items compared with others [16,41]. Compared with previous studies that compared FFQs with WFR, our results showed strong agreement for red meat (*r*=0.88) intake and fish and fish products, which are often consumed less frequently than other groups. A possible reason could be the differences in diets consumed in the Gulf compared with Western countries [47] and may be because of the short interval between the administration of the FFQ and the four-day WFR. 

### Strengths and Limitations

This study had many strengths, which included the comparison of the EatWellQ8 FFQ with 2 frequently used methods for dietary collection, one of them being the gold standard (a WFR) and the other a PFFQ to assess the reproducibility and relative validity of the EatWellQ8 FFQ [16]. Moreover, the sample size in this validation study was found to be adequate and comparable with the sample size used in previous studies [16,28,43,48].

Limitations of the validation study include the short interval time between administrations of the comparison tools (WFR and PFFQ) and the EatWellQ8 FFQ administration (7-10 days), which may have resulted in the similarity of responses between the tool. The addition of composite Kuwaiti dishes to the EatWellQ8 FFQ may have led to double reporting of food items and overestimation of caloric intake. In addition, the relatively large number of items (n=146) in the EatWellQ8 FFQ may have led to increased confusion and questionnaire fatigue or boredom. However, the validated comparison tools used (WFR and PFFQ) may also have been considered time consuming and burdensome for participants as the PFFQ, which included more than 200 items, takes approximately 35 to 40 minutes to complete, and the WFR required the weighing of food several times per day. Questionnaire tiring or boredom is particularly concerning as it can lead to underreporting of food items and may have therefore compromised the results [5]. However, the PFFQ was the only validated paper FFQ available for comparison in Kuwait, and WFR is a recommended comparison in validation studies.

Willet et al [49] suggested that the preferred sample size for FFQ validation studies is between 100 and 200, especially if they also take into account nutrient intakes; thus, the smaller sample size achieved in this study may be a limitation. However, it should be noted that the current trial did face recruitment issues. In addition, there was a high dropout rate in the trial, which may have resulted from the lack of an incentive upon completion or to participants’ unwillingness to complete all 3 aspects of the study because of fatigue or boredom. Owing to the limited data available on the participants (eg, lack of sociodemographic and habitual data), it was not possible to account for known issues in self-reporting or deduce whether the sample was representative of the Kuwaiti population.

An additional limitation is the lack of biomarker data or alternative objective reference measures to validate the subjective questionnaire. It should also be noted that the EatWellQ8 FFQ and nutrient assessments did not consider supplements in the calculation of nutrition intakes, which may have led to their inaccurate assessments. Although seasonality is a common limitation in validation studies, it was not a concern in the current trial as the period of assessment for both validation and reproducibility fell between fall and winter. Owing to the lack of recent food composition tables specific to Kuwait, an additional limitation may be inaccurate assessments of nutrient content of some food items, which further highlights the need for dietary assessment software that is specific for Kuwait.

### Conclusions

In conclusion, the web-based self-administered EatWellQ8 FFQ, developed to assess energy and nutrient intake in healthy adults living in Kuwait, was found to have good reproducibility and moderate relative validity compared with a PFFQ and a four-day WFR. The results indicate that the novel web-based FFQ could be used as a dietary intake tool for the assessment of dietary intake in healthy adults living in Kuwait.

## Appendix

Multimedia Appendix 1 Unadjusted correlation coefficients, cross-classification and spearman correlation coefficients of quartiles of mean energy, nutrients and food group intakes from repeat measures of the web-based EatWellQ8 FFQ, paper-based food frequency questionnaire, and weighed food record.

Multimedia Appendix 2 EatWellQ8 food frequency questionnaire food list.

## Abbreviations

%TE: percentage total energy
DDI: Dasman Diabetes Institute
EPIC: European Prospective Investigation of Cancer
FA: fatty acid
FFQ: food frequency questionnaire
LOA: limits of agreement
MUFA: monounsaturated fatty acid
Na: sodium
NCD: noncommunicable disease
PFFQ: paper-based food frequency questionnaire
PN: personalized nutrition
SCC: Spearman correlation coefficient
SFA: saturated fatty acid
WFR: weighed food record
