# Supplementary material for: Web-Based Dietary Intake Estimation to Assess the Reproducibility and Relative Validity of the EatWellQ8 Food Frequency Questionnaire: Validation Study
Source: JMIR Form Res. 2021 Mar 2;5(3):e13591. doi: 10.2196/13591 (PMC7967232; doi:10.2196/13591)
Supplement: Multimedia Appendix 1 [file formative_v5i3e13591_app1.docx]

Multimedia Appendix 2 Table 1. EatWellQ8 FFQ food list

| id | english-name | arabic-name | Food group |
| --- | --- | --- | --- |
| 0 | Broccoli, green leafy vegetables | القرنبيط | vegetables |
| 1 | Cabbage | ملفوف | vegetables |
| 2 | Carrot | جزر | vegetables |
| 3 | Cauliflower | زهرة | vegetables |
| 4 | beetroot | شمندر | vegetables |
| 5 | Cucumber | خيار | vegetables |
| 6 | Garlic | ثوم | vegetables |
| 7 | Sweet pepper | فلفل حلو | vegetables |
| 8 | peas | بزاليا | vegetables |
| 9 | Asparagus | هليون | vegetables |
| 10 | green beans | فاصوليا خضراء | vegetables |
| 11 | Onions | بصل | vegetables |
| 12 | Spinach | السبانخ | vegetables |
| 13 | Olive | زيتون | vegetables |
| 14 | Tomatoes | طماطم | vegetables |
| 15 | Corn | ذرة | vegetables |
| 16 | Mushroom | مشروم | vegetables |
| 17 | green salad | سلطة خضرة | vegetables |
| 18 | Avocado | أفوكادو | vegetables |
| 19 | Apple | تفاح | fruits |
| 20 | Banana | الموز | fruits |
| 21 | Dates | تمر | fruits |
| 22 | Grapes | عنب | fruits |
| 23 | Guava | جوافة | fruits |
| 24 | Kiwi | كيوي | fruits |
| 25 | Mango | مانجو | fruits |
| 26 | Melon | شمام | fruits |
| 27 | Tangerine,Orange | أفندي ,برتقال | fruits |
| 28 | Berries | أنواع التوت | fruits |
| 29 | Plum, Peach | قوجة ,خوخ | fruits |
| 30 | Pears | كمثرى | fruits |
| 31 | Pomegranate | رمان | fruits |
| 32 | Watermelon | بطيخ | fruits |
| 33 | Tinned Fruit | فاكهه معلبة | fruits |
| 34 | Brown Toast | توست أسمر | breads |
| 35 | White toast | توست أبيض | breads |
| 36 | Irani bread | خبز ايراني | breads |
| 37 | Lebnani bread brown | خبز لبناني أسمر | breads |
| 38 | Lebanese bread thin brown | خبز لبناني أسمر خفيف | breads |
| 39 | lebanese bread thin white | خبز لبناني أبيض خفيف | breads |
| 40 | Lebnani bread-white | خبز لبناني أبيض | breads |
| 41 | Bread roll | صمون | breads |
| 42 | Baqsam | بقصم | breads |
| 43 | Shaboora | شابوره | breads |
| 44 | sweetened cereal | كورن فليكس حالي | cereals |
| 45 | Breakfast wholegrain | كورن فليكس كامل الحبوب | cereals |
| 46 | Breakfast cereal | كورن فليكس | cereals |
| 47 | Muesli, oats | شوفان | cereals |
| 48 | White rice | أرز أبيض | potatoes |
| 49 | Brown rice | أرز أسمر | potatoes |
| 50 | Potatoes,boiled | بطاطا مسلوقة | potatoes |
| 51 | Fried potatoes | بطاطا مقلية | potatoes |
| 52 | Pasta | باستا | potatoes |
| 53 | Lasagne, Bashamel | لازانيا, بشاميل | potatoes |
| 54 | Spaghetti | معكرونة | potatoes |
| 55 | Spring roles | سبرينج ر ول | potatoes |
| 56 | Pizza | بيتزا | potatoes |
| 57 | Machbous laham | مجبوس لحم | kuwait |
| 58 | Machbous Deyay | مجبوس دجاج | kuwait |
| 59 | Vegetable beryani | برياني خضار | kuwait |
| 60 | Mourkokah | مرقوقه | kuwait |
| 61 | Biryani chicken | برياني دجاج | kuwait |
| 62 | Biryani | برياني لحم | kuwait |
| 63 | mourabyan | مربين | kuwait |
| 64 | Mowash Robyan | موش ربيان | kuwait |
| 65 | Meaddas | معدس | kuwait |
| 66 | Jareesh | جريش | kuwait |
| 67 | Harrees | هريس | kuwait |
| 68 | Motabak samak | مطبق سمج | kuwait |
| 69 | tomato sauce | دقوس | kuwait |
| 70 | Marag | مرق | kuwait |
| 71 | Orkra stew | مرق بامية | kuwait |
| 72 | Nakhi | نخي | kuwait |
| 73 | lentil soup | مرق عدس | kuwait |
| 74 | Falafil | فلافل | kuwait |
| 75 | stuffed vine leaves | ورق عنب | kuwait |
| 76 | Hommus | حمص | kuwait |
| 77 | Fool | فول مدمس | kuwait |
| 78 | Fatayer | فطاير | kuwait |
| 79 | Shawerma | شاويرما | kuwait |
| 80 | Fried fish | سمك مقلي | meat |
| 81 | Non smoked oily fish | سمك غني بالزيت غير مدخن | meat |
| 82 | Non Smoked oily fish canned | سمك غير مدخن معلب | meat |
| 83 | Smoked fish | سمك مدخن | meat |
| 84 | Shell fish | مأكولات بحريه | meat |
| 85 | Sushi | سوشي | meat |
| 86 | beef steak grilled | لحم مشوي | meat |
| 87 | Chicken | دجاج | meat |
| 88 | Fried Chicken | دجاج مقلي | meat |
| 89 | Deli meats | لحوم بارده | meat |
| 90 | burger | همبرجر | meat |
| 91 | Sausages | سوسج | meat |
| 92 | Kufta, Kubba | كفتة, كبة | meat |
| 93 | Milk, Laban | حليب أو لبن كامل الدسم | dairy |
| 94 | Low fat milk, Laban | حليب أو لبن قليل الدسم | dairy |
| 95 | Skimmed milk, laban | حليب أو لبن خالي الدسم | dairy |
| 96 | Condensed milk | حليب مركز حالي | dairy |
| 97 | Flavoured milk | حليب بالشوكولاته, فراولة | dairy |
| 98 | Soya, almond milk | حليب الصويا, اللوز | dairy |
| 99 | Fruit yogurt | روب بالفاكهه | dairy |
| 100 | Yogurt | روب كامل الدسم | dairy |
| 101 | Yogurt Low fat | روب قليل الدسم | dairy |
| 102 | High fat cheese | جبن غني الدهون ,الجيدر | dairy |
| 103 | Medium fat cheese | جبن متوسط الدسم ,الحلوم | dairy |
| 104 | Cream cheese | جبن الكريمي | dairy |
| 105 | Low fat cheese | جبن قليل الدسم , قريش | dairy |
| 106 | Lebneh | لبنه | dairy |
| 107 | Eggs, boiled | بيض مسلوق | dairy |
| 108 | Eggs Scrambelled | بيض مقلي | dairy |
| 109 | Salad cream | صلصه السلطه الكريميه | dairy |
| 110 | Other salad dressing | صلصه السلطه أنواع أخرى | dairy |
| 111 | Butter | الزبده | dairy |
| 112 | Margarine | المارجرين | dairy |
| 113 | Olive oil | زيبت الزيتون | dairy |
| 114 | Other vegetable oil | زيت نباتي أخر | dairy |
| 115 | Sweets biscuits | بسكوت حالي | sweets |
| 116 | croissants | كرواسون | sweets |
| 117 | Chocolates squares | قطعة شوكولاتة | sweets |
| 118 | Chocolate bars | شريط شوكولاته | sweets |
| 119 | Ice cream, ice lollies | ايس كريم | sweets |
| 120 | plain cake | كيك | sweets |
| 121 | Jelly | جيلي | sweets |
| 122 | Mamoul | معمول | sweets |
| 123 | muffins, donuts | مافين, دونت | sweets |
| 124 | Rich cakes | كيك مع الشوكولاته | sweets |
| 125 | pancakes, crepes | وافلز، بانكيك | sweets |
| 126 | Sweets , toffee | حلاو | sweets |
| 127 | Konafa | كنافه | sweets |
| 128 | lgaimat | حلو اللقيمات | sweets |
| 129 | Sugar added to coffee, tea | سكر مضاف للشاي و القهوه | sweets |
| 130 | Nut or chocolate spreads | كريمة الكاكاو والبندق | sweets |
| 131 | Nuts | المكسرات | sweets |
| 132 | Chips | بطاط | sweets |
| 133 | Jam, honey | مربى, عسل | sweets |
| 134 | Pickles, ketchup | مخلل, كاشب | sweets |
| 135 | Tea black, green, herbal | شاي الأحمر، الأخضر، أعشاب | drinks |
| 136 | Coffee | القهوه | drinks |
| 137 | Cappucino, latte etc | كابوشينو, لاتي الخ | drinks |
| 138 | Hot chocolate | حليب بالكاكاو الساخن | drinks |
| 139 | Pure fruit juice | عصير طبيعي | drinks |
| 140 | Fizzy drinks | مشروبات غازيه | drinks |
| 141 | Diet fizzy drinks | مشروبات غازيه دايت | drinks |
| 142 | Juice drinks | عصير بالنكتار | drinks |
| 143 | Creamy soups | شوربه بالكريمه | drinks |
| 144 | Non-creamy soups | شوربه خفيفه دون الكريمه | drinks |
| 145 | water | ماء | drinks |
