# Supplementary material for: Web-Based Dietary Intake Estimation to Assess the Reproducibility and Relative Validity of the EatWellQ8 Food Frequency Questionnaire: Validation Study
Source: JMIR Form Res. 2021 Mar 2;5(3):e13591. doi: 10.2196/13591 (PMC7967232; doi:10.2196/13591)
Supplement: Multimedia Appendix 2 [file formative_v5i3e13591_app2.docx]

## Multimedia Appendix 1:

##

**Table S1.**Unadjusted correlation coefficients and cross-classification of quartiles of mean energy and nutrient intakes derived from repeat measures of the web-based EatWellQ8 FFQ (n=53)

| Nutrient | Correlation | 95%CI | Quartiles, % Exact agreement^b^ | Exact agreement plus adjacent^c^ | Disagreement^d^ | Extreme disagreement^e^ |
| --- | --- | --- | --- | --- | --- | --- |
|  |  |  |  |  |  |  |
| Energy (kcal) | 0.79 ^a^ | (0.66;0.87) | 51 | 100 | 0 | 0 |
| Total Fat (g) | 0.75 ^a^ | (0.60;0.84) | 53 | 87 | 13 | 0 |
| Total Fat (% TE) | 0.42 ^a^ | (0.16;0.67) | 40 | 81 | 15 | 3 |
| SFA (g) | 0.68 ^a^ | (0.50;0.80) | 51 | 92 | 7 | 0 |
| SFA (% TE) | 0.53 ^a^ | (0.30;0.70) | 47 | 81 | 11 | 7 |
| MUFA (g) | 0.77 ^a^ | (0.63;0.86) | 53 | 92 | 5 | 2 |
| MUFA (% TE) | 0.49 ^a^ | (0.25;0.67) | 40 | 77 | 15 | 2 |
| PUFA (g) | 0.69 ^a^ | (0.52;0.81) | 53 | 91 | 5 | 3 |
| PUFA (% TE) | 0.37 ^a^ | (0.11;0.58) | 36 | 74 | 19 | 8 |
| Omega 3 (g) | 0.44 ^a^ | (0.20;0.63) | 51 | 83 | 13 | 3 |
| Protein (g) | 0.74 ^a^ | (0.58;0.84) | 55 | 91 | 7 | 2 |
| Protein (% TE) | 0.74 ^a^ | (0.58;0.84) | 42 | 92 | 3 | 2 |
| Carbohydrate (g) | 0.78 ^a^ | (0.64;0.86) | 57 | 94 | 5 | 0 |
| Carbohydrate (% TE) | 0.54 ^a^ | (0.31;0.70) | 42 | 83 | 21 | 0 |
| Total sugars (g) | 0.77 ^a^ | (0.63;0.86) | 53 | 94 | 5 | 0 |
| Total sugars (%TE) | 0.63 ^a^ | (0.43;0.76) | 42 | 87 | 13 | 0 |
| Calcium (mg) | 0.76 ^a^ | (0.61;0.85) | 45 | 94 | 5 | 0 |
| Total folate (µg) | 0.81 ^a^ | (0.69;0.88) | 62 | 92 | 7 | 0 |
| Iron (mg) | 0.82 ^a^ | (0.70;0.89) | 55 | 92 | 3 | 0 |
| Total carotene (µg) | 0.38 ^a^ | (0.12;0.58) | 57 | 94 | 5 | 0 |
| Riboflavin (mg) | 0.75 ^a^ | (0.60;0.84) | 55 | 92 | 7 | 0 |
| Thiamin (mg) | 0.76 ^a^ | (0.61;0.85) | 57 | 92 | 5 | 2 |
| Vitamin B6 (mg) | 0.69 ^a^ | (0.51;0.81) | 53 | 85 | 15 | 0 |
| Vitamin B12 (µg) | 0.66 ^a^ | (0.47;0.78) | 47 | 87 | 9 | 3 |
| Vitamin C (mg) | 0.75 ^a^ | (0.60;0.84) | 49 | 87 | 13 | 0 |
| Vitamin A RE (µg) | 0.42 ^a^ | (0.17;0.62) | 60 | 98 | 2 | 0 |
| Retinol (µg) | 0.57 ^a^ | (0.35;0.73) | 51 | 87 | 11 | 2 |
| Vitamin D (µg) | 0.63 ^a^ | (0.43;0.76) | 45 | 81 | 13 | 5 |
| Vitamin E (mg) | 0.81 ^a^ | (0.69;0.89) | 57 | 94 | 5 | 0 |
| Na (mg) | 0.75 ^a^ | (0.60;0.84) | 51 | 92 | 7 | 0 |

^a^ *P*<0.01

^b^ Exact agreement, % of case cross-classified into the same quartile.

^c^ Exact agreement plus adjacent, % of cases cross-classified into the same or adjacent quartile.

^d^ Disagreement, % of cases cross-classified 2 quartiles apart.

^e^ Extreme disagreement, % of cases cross-classified into extreme quartiles

%TE= Percentage Total Energy, SFA= Saturated Fatty Acids, MUFA= Monounsaturated Fatty Acids, PUFA= Polyunsaturated Fatty Acids, RE= Retinol Equivalent, Na= Sodium, CI=Confidence Interval

**Table S2**. Spearman correlation coefficients (SCC) and cross-classification of quartiles of food group intake derived from repeat measures of the web-based EatWellQ8 FFQ (n=53)

| Nutrient | Correlation ^a^ | 95% CI | Quartiles,% Exact agreement^b^ | Exact agreement plus adjacent^c^ | Disagreement^d^ | Extreme disagreement^e^ |
| --- | --- | --- | --- | --- | --- | --- |
| Rice, pasta, grains and starches | 0.57 | (0.35;0.73) | 51 | 86 | 11 | 2 |
| Savories (lasagne, pizza) | 0.40 | (0.14;0.60) | 49 | 75 | 16 | 7 |
| White bread (rolls, tortillas, crackers) | 0.67 | (0.48;0.79) | 60 | 83 | 9 | 7 |
| Wholegrain and brown breads and rolls | 0.56 | (0.34;0.72) | 57 | 77 | 16 | 5 |
| Breakfast cereals and porridge | 0.76 | (0.61;0.85) | 67 | 91 | 7 | 2 |
| Biscuits | 0.58 | (0.36;0.73) | 63 | 83 | 10 | 6 |
| Cakes, pastries and buns | 0.80 | (0.67;0.88) | 66 | 91 | 8 | 0 |
| Milk | 0.72 | (0.56;0.83) | 58 | 90 | 10 | 0 |
| Cheeses | 0.51 | (0.28;0.68) | 58 | 85 | 10 | 5 |
| Yoghurts | 0.59 | (0.38;0.74) | 48 | 80 | 15 | 2 |
| Ice cream, creams and desserts | 0.79 | (0.66;0.87) | 68 | 91 | 6 | 2 |
| Eggs and egg dishes | 0.84 | (0.73;0.90) | 71 | 96 | 3 | 0 |
| Fats and oils (eg,butter, low-fat spreads, hard cooking fats) | 0.83 | (0.72;0.89) | 65 | 95 | 3 | 2 |
| Potatoes and potato dishes, Chipped, fried and roasted potatoes | 0.88 | (0.80;0.92) | 75 | 93 | 6 | 0 |
| Peas, beans and lentils and vegetable and pulse dishes | 0.82 | (0.70;0.89) | 73 | 91 | 5 | 3 |
| Green vegetables | 0.67 | (0.49;0.79) | 63 | 81 | 18 | 0 |
| Carrots | 0.75 | (0.60;0.84) | 66 | 93 | 3 | 3 |
| Salad vegetables (e.g., lettuce) | 0.64 | (0.45;0.77) | 45 | 85 | 11 | 3 |
| Other vegetables (e.g., onions) | 0.65 | (0.46;0.78) | 63 | 88 | 3 | 8 |
| Tinned fruit or vegetables | 0.70 | (0.53;0.81) | 53 | 83 | 13 | 3 |
| Bananas | 0.81 | (0.69;0.88) | 71 | 93 | 5 | 2 |
| Other fruits (e.g., apples, pears, oranges) | 0.76 | (0.61;0.85) | 71 | 88 | 11 | 0 |
| Nuts and seeds, herbs and spices | 0.71 | (0.54;0.82) | 68 | 86 | 8 | 5 |
| Fish and fish products/dishes | 0.76 | (0.61;0.85) | 65 | 90 | 6 | 3 |
| Red meat (e.g., beef, veal, lamb, etc.) | 0.85 | (0.75;0.91) | 70 | 90 | 8 | 0 |
| Poultry (chicken and turkey) | 0.59 | (0.38;0.74) | 53 | 83 | 13 | 3 |
| Meat products (e.g., burgers, sausages, pies, processed meats) | 0.93 | (0.88;0.95) | 76 | 98 | 2 | 0 |
| Sugars, syrups, preserves and sweeteners | 0.46 | (0.22;0.64) | 53 | 76 | 11 | 11 |
| Confectionary, savory snacks | 0.37 | (0.11;0.58) | 46 | 66 | 23 | 10 |
| Soups, sauces, miscellaneous foods | 0.64 | (0.45;0.77) | 61 | 83 | 10 | 6 |
| Teas and coffees | 0.63 | (0.43;0.76) | 48 | 88 | 10 | 2 |
| Fruit Juice and Fizzy Drinks | 0.43 | (0.18;0.62) | 45 | 68 | 20 | 11 |

^a^ *P*<0.01

^b^ Exact agreement, % of case cross-classified into the same quartile.

^c^ Exact agreement plus adjacent, % of cases cross-classified into the same or adjacent quartile.

^d^ Disagreement, % of cases cross-classified 2 quartiles apart.

^e^ Extreme disagreement, % of cases cross-classified into extreme quartiles.

CI= Confidence Interval

**Table S3**. Unadjusted correlation coefficients and cross-classification of quartiles of mean energy and nutrient intakes derived from comparative measures of a PFFQ to the web-based EatWellQ8 FFQ (n=46)

| Nutrient | Correlation | 95% CI | Quartiles, % Exact agreement^b^ | Exact agreement plus adjacent^c^ | Disagreement^d^ | Extreme disagreement^e^ |
| --- | --- | --- | --- | --- | --- | --- |
| Energy (kcal) | 0.73 ^a^ | (0.56;0.84) | 53 | 93 | 7 | 0 |
| Total Fat (g) | 0.59 ^a^ | (0.36;0.75) | 35 | 87 | 13 | 0 |
| Total Fat (% TE) | 0.45 ^a^ | (0.18;0.65) | 48 | 76 | 24 | 0 |
| SFA (g) | 0.68 ^a^ | (0.48;0.81) | 46 | 93 | 4 | 0 |
| SFA (% TE) | 0.49 ^a^ | (0.23;0.68) | 52 | 83 | 17 | 0 |
| MUFA (g) | 0.62 ^a^ | (0.40;0.77) | 39 | 83 | 11 | 0 |
| MUFA (% TE) | 0.44 ^a^ | (0.17;0.64) | 50 | 80 | 15 | 4 |
| PUFA (g) | 0.64 ^a^ | (0.43;0.78) | 52 | 85 | 15 | 0 |
| PUFA (% TE) | 0.58 ^a^ | (0.35;0.74) | 35 | 91 | 9 | 0 |
| Omega 3 (g) | 0.19 | (-0.11;0.45) | 24 | 63 | 28 | 9 |
| Protein (g) | 0.59 ^a^ | (0.36;0.75) | 48 | 83 | 17 | 0 |
| Protein (% TE) | 0.57 ^a^ | (0.34;0.74) | 48 | 87 | 9 | 4 |
| Carbohydrate (g) | 0.66 ^a^ | (0.45;0.79) | 56 | 85 | 11 | 4 |
| Carbohydrate (% TE) | 0.51 ^a^ | (0.26;0.70) | 48 | 83 | 13 | 4 |
| Total sugars (g) | 0.59 ^a^ | (0.36;0.75) | 54 | 87 | 11 | 2 |
| Total sugars (%TE) | 0.45 ^a^ | (0.18;0.65) | 50 | 80 | 15 | 4 |
| Calcium (mg) | 0.63 ^a^ | (0.42;0.78) | 52 | 87 | 13 | 0 |
| Total folate (µg) | 0.51 ^a^ | (0.26;0.70) | 37 | 83 | 15 | 2 |
| Iron (mg) | 0.46 ^a^ | (0.20;0.66) | 39 | 78 | 20 | 2 |
| Total carotene (µg) | 0.59 ^a^ | (0.36;0.75) | 46 | 85 | 11 | 4 |
| Riboflavin (mg) | 0.71 ^a^ | (0.53;0.83) | 48 | 91 | 9 | 0 |
| Thiamin (mg) | 0.63 ^a^ | (0.42;0.78) | 48 | 87 | 13 | 0 |
| Vitamin B6 (mg) | 0.65 ^a^ | (0.44;0.79) | 48 | 83 | 17 | 0 |
| Vitamin B12 (µg) | 0.47 ^a^ | (0.21;0.67) | 43 | 83 | 11 | 7 |
| Vitamin C (mg) | 0.51 ^a^ | (0.26;0.70) | 35 | 89 | 4 | 7 |
| Vitamin A RE (µg) | 0.51 ^a^ | (0.26;0.70) | 48 | 83 | 11 | 7 |
| Retinol (µg) | 0.27 | (-0.02;0.52) | 35 | 76 | 13 | 11 |
| Vitamin D (µg) | 0.42 ^a^ | (0.15;0.63) | 39 | 76 | 22 | 2 |
| Vitamin E (mg) | 0.62 ^a^ | (0.40;0.77) | 50 | 87 | 13 | 0 |
| Na (mg) | 0.55 ^a^ | (0.22;0.76) | 57 | 85 | 11 | 4 |

^a^ *P*<0.01

^b^ Exact agreement, % of case cross-classified into the same quartile.

^c^ Exact agreement plus adjacent, % of cases cross-classified into the same or adjacent quartile.

^d^ Disagreement, % of cases cross-classified 2 quartiles apart.

^e^ Extreme disagreement, % of cases cross-classified into extreme quartiles

%TE= Percentage Total Energy, SFA= Saturated Fatty Acids, MUFA= Monounsaturated Fatty Acids, PUFA= Polyunsaturated Fatty Acids,RE= Retinol Equivalent, Na= Sodium

**Table S4**. Spearman correlation coefficients (SCC) and cross-classification of quartiles of food group intake derived from the web-based EatWellQ8 FFQ and PFFQ (n=46)

| Nutrient | Correlation ^a^ | 95% CI | Quartiles,% Exact agreement^c^ | Exact agreement plus adjacent^d^ | Disagreement^e^ | Extreme disagreement^f^ |
| --- | --- | --- | --- | --- | --- | --- |
| Rice, pasta, grains and starches | 0.29 ^b^ | (0.01;0.54) | 37 | 73 | 17 | 9 |
| Savories (lasagne, pizza) | 0.32^b^ | (0.03;0.56) | 39 | 76 | 20 | 4 |
| White bread (rolls, tortillas, crackers) | 0.33 ^b^ | (0.04;0.57) | 24 | 78 | 11 | 11 |
| Wholegrain and brown breads and rolls | 0.43^a^ | (0.16;0.64) | 50 | 71 | 17 | 7 |
| Breakfast cereals and porridge | 0.39^a^ | (0.11;0.61) | 54 | 80 | 9 | 11 |
| Biscuits | 0.34 ^b^ | (0.06;0.57) | 43 | 76 | 11 | 13 |
| Cakes, pastries and buns | 0.41^a^ | (0.14;0.63) | 37 | 74 | 19 | 7 |
| Milk | 0.36 ^b^ | (0.08;0.59) | 39 | 72 | 22 | 7 |
| Cheeses | 0.50 ^a^ | (0.24;0.69) | 45 | 74 | 15 | 4 |
| Yoghurts | 0.34 ^b^ | (0.06;0.57) | 35 | 76 | 11 | 13 |
| Ice cream, creams and desserts | 0.39 ^a^ | (0.11;0.61) | 48 | 65 | 26 | 8 |
| Eggs and egg dishes | 0.44 ^a^ | (0.17;0.64) | 47 | 80 | 11 | 8 |
| Fats and oils (eg,butter, low-fat spreads, hard cooking fats) | 0.45 ^a^ | (0.18;0.71) | 50 | 76 | 17 | 7 |
| Potatoes and potato dishes, Chipped, fried and roasted potatoes | 0.32 ^b^ | (0.03;0.56) | 40 | 73 | 13 | 13 |
| Peas, beans and lentils and vegetable and pulse dishes | 0.37 ^b^ | (0.10;0.60) | 52 | 80 | 13 | 7 |
| Green vegetables | 0.31 ^b^ | (0.02;0.55) | 35 | 78 | 11 | 11 |
| Carrots | 0.32 ^b^ | (0.03;0.56) | 41 | 69 | 17 | 13 |
| Salad vegetables (e.g., lettuce) | 0.37 ^b^ | (0.10;0.60) | 37 | 65 | 32 | 2 |
| Other vegetables (e.g., onions) | 0.37 ^b^ | (0.10;0.60) | 37 | 73 | 15 | 11 |
| Tinned fruit or vegetables | 0.35 ^b^ | (0.67;0.58) | 41 | 73 | 11 | 15 |
| Bananas | 0.51 ^a^ | (0.26;0.69) | 50 | 84 | 4 | 9 |
| Other fruits (e.g., apples, pears, oranges) | 0.42 ^a^ | (0.15;0.68) | 32 | 82 | 13 | 4 |
| Nuts and seeds, herbs and spices | 0.30 ^b^ | (0.01;0.54) | 39 | 69 | 17 | 13 |
| Fish and fish products/dishes | 0.22 | (-0.07;0.47) | 54 | 69 | 11 | 20 |
| Red meat (e.g., beef, veal, lamb, etc.) | 0.36 ^b^ | (0.07;0.58) | 37 | 71 | 21 | 7 |
| Poultry (chicken and turkey) | 0.40 ^a^ | (0.13;0.62) | 50 | 70 | 21 | 8 |
| Meat products (e.g., burgers, sausages, pies, processed meats) | 0.41 ^a^ | (0.14;0.63) | 47 | 71 | 17 | 11 |
| Sugars, syrups, preserves and sweeteners | 0.34 ^b^ | (0.06;0.57) | 56 | 70 | 17 | 13 |
| Confectionary, savory snacks | 0.39 ^a^ | (0.11;0.61) | 43 | 78 | 9 | 13 |
| Soups, sauces, miscellaneous foods | 0.42 ^a^ | (0.15;0.68) | 60 | 76 | 11 | 13 |
| Teas and coffees | 0.43 ^a^ | (0.16;0.64) | 37 | 82 | 13 | 4 |
| Fruit Juice and Fizzy Drinks | 0.42 ^a^ | (0.15;0.68) | 52 | 80 | 9 | 13 |

^a^ *P*<0.01

^b^ *P*<0.05

^c^ Exact agreement, % of case cross-classified into the same quartile.

^d^ Exact agreement plus adjacent, % of cases cross-classified into the same or adjacent quartile.

^e^ Disagreement, % of cases cross-classified 2 quartiles apart.

^f^ Extreme disagreement, % of cases cross-classified into extreme quartiles.

**Table S5.** Unadjusted correlation coefficients and cross-classification of quartiles of mean energy and nutrient intakes derived from comparative measures of a 4-day WFR (N=46) to the web-based EatWellQ8 FFQ

| Nutrient | Correlation | 95% CI | Quartiles, % Exact agreement^b^ | Exact agreement plus adjacent^c^ | Disagreement^d^ | Extreme disagreement^e^ |
| --- | --- | --- | --- | --- | --- | --- |
| Energy (kcal) | 0.88 ^a^ | (0.79;0.93) | 67 | 93 | 6 | 0 |
| Total Fat (g) | 0.69 ^a^ | (0.50;0.82) | 52 | 84 | 4 | 2 |
| Total Fat (% TE) | 0.47 ^a^ | (0.21;0.67) | 37 | 80 | 15 | 4 |
| SFA (g) | 0.80 ^a^ | (0.67;0.88) | 47 | 93 | 4 | 2 |
| SFA (% TE) | 0.56 ^a^ | (0.32;0.73) | 37 | 84 | 15 | 0 |
| MUFA (g) | 0.52 ^a^ | (0.27;0.70) | 45 | 78 | 19 | 2 |
| MUFA (% TE) | 0.45 ^a^ | (0.18;0.65) | 41 | 71 | 26 | 2 |
| PUFA (g) | 0.49 ^a^ | (0.23;0.68) | 28 | 84 | 10 | 4 |
| PUFA (% TE) | 0.55 ^a^ | (0.31;0.72) | 45 | 84 | 13 | 2 |
| Omega 3 (g) | 0.49 ^a^ | (0.23;0.68) | 50 | 80 | 13 | 2 |
| Protein (g) | 0.81 ^a^ | (0.68;0.89) | 58 | 93 | 7 | 0 |
| Protein (% TE) | 0.57 ^a^ | (0.34;0.74) | 35 | 89 | 9 | 2 |
| Carbohydrate (g) | 0.72 ^a^ | (0.54;0.84) | 65 | 91 | 8 | 0 |
| Carbohydrate(%TE) | 0.70 ^a^ | (0.52;0.82) | 45 | 89 | 10 | 0 |
| Total sugars (g) | 0.72 ^a^ | (0.54;0.84) | 56 | 91 | 8 | 0 |
| Total sugar (%TE) | 0.57 ^a^ | (0.34;0.74) | 41 | 95 | 6 | 2 |
| Calcium (mg) | 0.74 ^a^ | (0.57;0.85) | 52 | 91 | 8 | 0 |
| Total folate (µg) | 0.68 ^a^ | (0.49;0.81) | 63 | 89 | 11 | 0 |
| Iron (mg) | 0.40 ^a^ | (0.13;0.62) | 32 | 80 | 10 | 8 |
| Total carotene (µg) | 0.53 ^a^ | (0.28;0.71) | 46 | 80 | 15 | 4 |
| Riboflavin (mg) | 0.68 ^a^ | (0.49;0.81) | 54 | 89 | 10 | 0 |
| Thiamin (mg) | 0.58 ^a^ | (0.35;0.74) | 46 | 83 | 15 | 2 |
| Vitamin B6 (mg) | 0.59 ^a^ | (0.36;0.75) | 41 | 78 | 19 | 2 |
| Vitamin B12 (µg) | 0.73 ^a^ | (0.56;0.84) | 56 | 91 | 9 | 0 |
| Vitamin C (mg) | 0.76 ^a^ | (0.60;0.86) | 57 | 91 | 8 | 0 |
| Vitamin A RE(µg) | 0.57 ^a^ | (0.34;0.74) | 43 | 83 | 17 | 0 |
| Retinol (µg) | 0.40 ^a^ | (0.13;0.62) | 39 | 82 | 9 | 9 |
| Vitamin D (µg) | 0.50 ^a^ | (0.25;0.69) | 45 | 78 | 19 | 2 |
| Vitamin E (mg) | 0.64 ^a^ | (0.43;0.78) | 41 | 93 | 2 | 4 |
| Na (mg) | 0.55^a^ | (0.31;0.72) | 52 | 83 | 17 | 0 |

a *P*<.01

b Exact agreement, % of case cross-classified into the same quartile.

c Exact agreement plus adjacent, % of cases cross-classified into the same or adjacent quartile.

d Disagreement, % of cases cross-classified 2 quartiles apart.

e Extreme disagreement, % of cases cross-classified into extreme quartiles.

%TE= Percentage Total Energy, SFA= Saturated Fatty Acids, MUFA= Monounsaturated Fatty Acids, PUFA= Polyunsaturated Fatty Acids, RE= Retinol Equivalent, Na= Sodium

**Table S6**. Spearman correlation coefficients (SCC) and cross-classification of quartiles of food group intake derived from the web-based EatWellQ8 FFQ and 4-day WFR (n=46)

| Nutrient | Correlation ^a^ | 95% CI | Quartiles,% Exact agreement^c^ | Exact agreement plus adjacent^d^ | Disagreement^e^ | Extreme disagreement^f^ |
| --- | --- | --- | --- | --- | --- | --- |
| Rice, pasta, grains and starches | 0.37^b^ | (0.10;60) | 37 | 80 | 15 | 4 |
| Savories (lasagne, pizza) | 0.45^a^ | (0.18;0.65) | 34 | 80 | 17 | 2 |
| White bread (rolls, tortillas, crackers) | 0.81^a^ | (0.68;0.89) | 60 | 91 | 9 | 0 |
| Wholegrain and brown breads and rolls | 0.74 ^a^ | (0.57;0.85) | 65 | 91 | 9 | 0 |
| Breakfast cereals and porridge | 0.36^b^ | (0.08;0.59) | 41 | 76 | 11 | 13 |
| Biscuits | 0.53^a^ | (0.28;0.71) | 39 | 80 | 13 | 7 |
| Cakes, pastries and buns | 0.64 ^a^ | (0.43;0.78) | 63 | 89 | 6 | 6 |
| Milk | 0.48 ^a^ | (0.22;0.67) | 45 | 84 | 7 | 9 |
| Cheeses | 0.84 ^a^ | (0.73;0.91) | 56 | 95 | 4 | 0 |
| Yoghurts | 0.36 ^b^ | (0.08;0.59) | 45 | 73 | 20 | 7 |
| Ice cream, creams and desserts | 0.33 ^b^ | (0.04;0.56) | 45 | 82 | 9 | 9 |
| Eggs and egg dishes | 0.76 ^a^ | (0.60;0.86) | 54 | 95 | 2 | 2 |
| Fats and oils (eg,butter, low-fat spreads, hard cooking fats) | 0.84 ^a^ | (0.73;0.91) | 58 | 93 | 7 | 0 |
| Potatoes and potato dishes, Chipped, fried and roasted potatoes | 0.76 ^a^ | (0.60;0.86) | 60 | 93 | 7 | 0 |
| Peas, beans and lentils and vegetable and pulse dishes | 0.79 ^a^ | (0.65;0.88) | 65 | 93 | 4 | 2 |
| Green vegetables | 0.31^b^ | (0.02;0.55) | 28 | 71 | 26 | 2 |
| Carrots | 0.72 ^a^ | (0.54;0.84) | 65 | 91 | 2 | 7 |
| Salad vegetables (e.g., lettuce) | 0.59 ^a^ | (0.36;0.75) | 43 | 84 | 13 | 2 |
| Other vegetables (e.g., onions) | 0.56 ^a^ | (0.32;0.73) | 67 | 84 | 7 | 9 |
| Tinned fruit or vegetables | 0.31 ^b^ | (0.02;0.55) | 43 | 74 | 17 | 9 |
| Bananas | 0.30 ^b^ | (0.01;0.54) | 45 | 71 | 13 | 15 |
| Other fruits (e.g., apples, pears, oranges) | 0.40 ^a^ | (0.13;0.62) | 43 | 78 | 24 | 4 |
| Nuts and seeds, herbs and spices | 0.66 ^a^ | (0.46;0.80) | 41 | 85 | 13 | 2 |
| Fish and fish products/dishes | 0.63 ^a^ | (0.42;0.78) | 54 | 91 | 4 | 4 |
| Red meat (e.g., beef, veal, lamb, etc.) | 0.88 ^a^ | (0.79;0.93) | 65 | 97 | 2 | 0 |
| Poultry (chicken and turkey) | 0.53 ^a^ | (0.29;0.71) | 50 | 84 | 13 | 2 |
| Meat products (e.g. ,burgers, sausages, pies, processed meats) | 0.37 ^b^ | (0.10;0.60) | 43 | 73 | 15 | 13 |
| Sugars, syrups, preserves and sweeteners | 0.43 ^a^ | (0.16;0.64) | 43 | 78 | 13 | 11 |
| Confectionary, savoury snacks | 0.30 ^b^ | (0.01;0.54) | 52 | 71 | 15 | 13 |
| Soups, sauces, miscellaneous foods | 0.57 ^a^ | (0.34;0.74) | 63 | 84 | 2 | 13 |
| Teas and coffees | 0.44 ^a^ | (0.17;0.65) | 43 | 76 | 21 | 2 |
| Fruit Juice and Fizzy Drinks | 0.61 ^a^ | (0.39;0.76) | 54 | 82 | 11 | 7 |

^a^ *P*<0.01

^b^ *P*<0.05

^c^ Exact agreement, % of case cross-classified into the same quartile.

^d^ Exact agreement plus adjacent, % of cases cross-classified into the same or adjacent quartile.

^e^ Disagreement, % of cases cross-classified 2 quartiles apart.

^f^ Extreme disagreement, % of cases cross-classified into extreme quartiles.

**CI= Confidence Interval**
